# Emergency gastrointestinal surgery in patients undergoing antithrombotic therapy in a single general hospital: a propensity score-matched analysis

**DOI:** 10.1186/s12876-021-01897-0

**Published:** 2021-08-21

**Authors:** Shinya Abe, Katsunori Ami, Akira Katsuno, Noriyasu Tamura, Toshiko Harada, Shunsuke Hamasaki, Yusuke Kitagawa, Taku Machida, Naoyuki Umetani

**Affiliations:** Department of Digestive Surgery, Kawakita General Hospital, 1-7-3 Kitaasagaya, Suginami-ku, Tokyo, 166-0001 Japan

**Keywords:** Antithrombotic therapy, Gastrointestinal surgery, Emergency surgery, Propensity score matched analysis

## Abstract

**Background:**

This study aimed to review and evaluate the surgical outcomes, particularly intraoperative severe blood loss and postoperative blood complications, of emergency gastrointestinal surgery in patients undergoing antithrombotic therapy (AT). Emergency surgeries for patients with antithrombotic medication have been increasing in the aging population. However, the effect of AT on intraoperative blood loss and perioperative complications remains unclear.

**Methods:**

We retrospectively reviewed 732 patients who underwent emergency gastrointestinal surgery between April 2014 and March 2019. Patients were classified into AT group and Non-AT group, and propensity score-matched analysis was performed to compare the short surgical outcomes between the groups. Additionally, risk factors in severe estimated blood loss (EBL) and postoperative bleeding complications were assessed.

**Results:**

Altogether, 64 patients received AT; 50 patients and 12, and 2 were given antiplatelet and anticoagulant, and both drugs, respectively. After propensity score matching, EBL (101 vs. 99 mL; *p* = 0.466) and postoperative complications (14 vs. 16 patients; *p* = 0.676) were similar between the groups (63 patients matched paired). Intraoperative severe bleeding (EBL ≥ 492 mL) occurred in 44 patients. Multivariate analysis using the full cohort revealed that antithrombotic drug use was not an independent risk factor for severe bleeding and postoperative bleeding complications.

**Conclusions:**

This study demonstrated antithrombotic drugs do not adversely affect the perioperative outcomes of emergency gastrointestinal surgery.

## Introduction

Medical science and technology improvements are leading to an increase in aging people patients with more comorbidities. Accordingly, the number of patients taking anticoagulant drugs or antiplatelet drugs to prevent primary and secondary thromboembolism from atrial fibrillation, heart-valve replacement, and coronary-artery stenting is increasing [1, 2].

In abdominal surgery, perioperative antithrombotic therapy (AT) may increase intraoperative blood loss and postoperative bleeding complications [3, 4]. Therefore, temporary cessation of antiplatelet and anticoagulant drugs is recommended before cardiac or non-cardiac surgery [5–7]. Hence, various perioperative AT management protocols have been developed in elective laparotomy and laparoscopic surgery, such as cholecystectomy, nephrectomy, colon resection, and rectal resection [8–11].

Emergency general surgery patients are well-known for facing high risks for postoperative complications and death within 30 postoperative days because of an extremely high prevalence of systemic inflammatory response syndrome, sepsis, and septic shock in this subpopulation [12–14]. Although vitamin K antagonizes warfarin, most antithrombotic drugs remain in development [15]. Surgeons, therefore, are faced with difficult choices such as whether to perform emergency surgeries in patients undergoing AT. There is accumulating evidence on emergency surgery for patients undergoing antiplatelet therapy for acute appendicitis and acute cholecystitis [16–18]. AT may have a greater effect in patients requiring emergency surgery than in those requiring elective surgery. However, peri- and postoperative outcomes in patients receiving AT remain unclear in an emergency gastrointestinal surgery.

This study aimed to review and evaluate the surgical outcomes, particularly intraoperative severe blood loss and postoperative blood complications, of emergency gastrointestinal surgery in patients undergoing antithrombotic therapy.

## Materials and methods

### Patient classification

We retrospectively evaluated 732 consecutive critical digestive disorder patients who underwent surgery within 24 h after medical reception between April 2014 and March 2019 at our institution. The operation criteria requiring emergency surgery were determined by the digestive surgeons’ opinion based on the laboratory data, computed tomography findings, the abdominal findings, and patients’ intention. We excluded patients who underwent surgery over 24 h from the hospital visit.

Patients were classified into the AT (antithrombotic therapy) group or the Non-AT group according to medication history for AT. AT is administering either or both of antiplatelet and anticoagulant drugs.

The study protocol was approved by the Ethics Committee at Kawakita General Hospital.

### Treatment and postoperative management of patients undergoing antithrombotic therapy

The surgical procedure for laparotomy or laparoscopy was decided on by an individual surgeon, and we had no strict selection criteria for emergency laparoscopic surgery. During the study period, the percentages of emergency laparoscopic surgery in the fiscal year from 2014 to 2018 constantly increased per year at 29%, 48%, 62%, 63%, and 75%, respectively.

In the patients taking anticoagulants, unfractionated heparin was postoperatively restarted immediately after surgery if there were no postoperative complications. Oral antithrombotic agents were also immediately resumed.

### Data collection

Patient data such as age, gender, postoperative diagnosis, Charlson comorbidity index, body mass index (BMI), American Society of Anesthesiologists classification of physical status (ASA・PS), and preoperative laboratory data including white blood cell, platelet counts, serum level of creatine, c-reactive protein (CRP), prothrombin time-international normalized ratio (PT(INR)), and activated partial thromboplastin time (APTT) were retrospectively retrieved from medical records. Postoperative complications were assessed using the Clavien-Dindo classification [19], with grade II or greater defined as significant. Data on surgical procedure, operating time, estimated blood loss, and perioperative blood transfusion were also obtained from the medical charts and records. Indication of blood transfusion was made with agreement of anesthesiologists and surgeons based on preoperative hemoglobin level, intraoperative vital signs, and intraoperative hemoglobin level. In our institution, the criteria of hemoglobin values were mostly 8 g/dL. Postoperative complications were defined as adverse events that occurred in 30 days after surgery.

### Statistical analysis

Categorized variables were assessed by using the chi-squared test or Fisher’s exact probability test, and continuous variables were assessed by using the Student’s test or Wilcoxon test. Propensity score matching was used to minimize the selection bias due to unbalanced baseline characteristics between patients in the AT and Non-AT groups for emergency gastrointestinal surgery [20]. The parameters that were different between both groups and other potential factors that could influence surgical outcomes such as age, gender, type of postoperative diagnosis, Charlson comorbidity index, and ASA・PS were selected to create a propensity score ranging from zero to one using a logistic regression model. Subsequently, we performed a 1:1 match between both groups, using the nearest neighbor matching with a caliper set to 0.2 of the standard deviation of the propensity score logit [21].

Variables with *p* value < 0.05 on univariate analysis were further evaluated using logistic regression analyses in multivariate analyses. All statistical analyses were performed using JMP software ver. Pro 11.0 (SAS Institute Inc., Cary, NC, USA). *P* values < 0.05 were considered statistically significant.

## Results

Altogether, 732 patients who underwent emergency gastrointestinal surgery for critical digestive disorders were analyzed during the study period (median age: 53 years old, 400 male patients (55%), mostly postoperative diagnosis: appendicitis (60%)). 64 (8.7%) patients were under antithrombotic therapy (AT) in the present study. We classified the patients under AT into 3 groups, such as antiplatelet (50 patients) or anticoagulant (12) or both drugs group (2), respectively. Because the risk of perioperative bleeding complications is probably different in each therapy. The patient profile on chronic antithrombotic medication is shown in Table 1. The most common reason for the antiplatelet use was stroke followed by ischemic heart disease. In contrast, atrial fibrillation was the leading cause for anticoagulant use. The breakdown of the antiplatelets was aspirin (30 patients), clopidogrel (14), cilostazol (5), ticlopidine (2) and dipyridamole (1), whereas that of the anticoagulants was warfarin (11 patients), apixaban (1), edoxaban (1) and dabigatran (1).

**Table 1:** Patient profile on chronic antithrombotic medication

	Antiplatelets	Anticoagulants	Both
Patient history or current comorbidities	(n = 50)	(n = 12)	(n = 2)
Stroke	23 (46%)	2 (17%)	0
Ischemic heart disease	20 (40%)	0	1 (50%)
Artificial fibrillation	1 (2%)	8 (67%)	1 (50%)
Peripheral artery disease	1 (2%)	1 (8%)	0
Cardiac valve replacement	1 (2%)	0	0
Deep vein thrombosis	0	1 (8%)	0
Central retinal artery occlusion	1 (2%)	0	0
Unknown	3 (6%)	0	0

Based on the hypothesis that advanced age patients with higher Charlson comorbidity index (CCI) scores frequently received antithrombotic therapy, we compared patients with advanced age and higher CCI to the other patients. Twenty-one patients were aged over 65 years and had a CCI of 3 or more in this cohort. Of these patients, two (9.5%) had received antithrombotic therapy compared to 62 in the other group. Although the number of patients was small, they had poor postoperative outcomes such as more blood loss (277 mL vs. 9 mL, p < 0.01), frequent blood transfusion (23% vs. 4.5%, p < 0.01), and frequent hospital death (19% vs. 2.9%, p < 0.01).

To examine the clinical impact of antithrombotic drugs on short postoperative outcomes, we used propensity score matching to balance baseline characteristics. The baseline characteristics of the patients in each group are summarized in Table 2. Before propensity score matching, it was observed that the AT group were older, had higher CCI, poor ASA・PS, and had a higher white blood cell count than the Non-AT group. The predominant kinds of postoperative diagnosis of the AT and the Non-AT group are small bowel obstruction, appendicitis, and upper gastrointestinal (UGI) perforation. Therefore, we should have adjusted a large number of differences of baseline characteristics between the AT and Non-AT groups to compare the short surgical outcomes for emergency gastrointestinal surgery. To minimize the selection bias due to unbalanced baseline characteristics between patients in the AT and Non-AT groups, we used propensity score matching using the parameters such as age, gender, type of postoperative diagnosis, CCI, and ASA・PS. Sixty-three matched pairs were created from the original cohort (Table 2).

**Table 2:** Baseline characteristics of patients in the cohort

Variables		n = 732	No-matched cohort			Matched cohort		
			AT (n = 64)	Non-AT (n = 668)	*p* value	AT (n = 63)	Non-AT (n = 63)	*p* value
*Patient-related factors*								
Age*	Years	51 (34–74)	83 (76–8)	47 (32–68)	< 0.001	83 (76–87)	81 (69–87)	0.214
Sex	Male	400 (55%)	39 (61%)	361 (54%)	0.290	39 (62%)	32 (51%)	0.209
Postoperative diagnosis	Appendicitis	441 (60%)	9 (14%)	432 (65%)	< 0.001	9 (14%)	10 (16%)	
	Small bowel obstruction	142 (19%)	33 (52%)	109 (16%)		33 (52%)	28 (44%)	
	UGI perforation	47 (6%)	8 (12%)	39 (6%)		8(13%)	9 (14%)	
	Cholecystitis	34 (5%)	3 (5%)	34 (5%)		3 (5%)	0	
	Large bowel perforation	30 (4%)	4 (6%)	31 (4%)		4 (6%)	7 (11%)	
	Large bowel obstruction	23 (3%)	2 (3%)	21 (3%)		2 (3%)	8 (13%)	
	Bacterial peritonitis	7 (1%)	1 (2%)	6 (1%)		1 (1%)	0	
	Gastrointestinal bleeding	6 (1%)	3 (5%)	3 (0.5%)		3 (5%)	1 (1%)	
	Others	2 (0.2%)	1 (0.2%)	1 (0.2%)				
Charlson comorbidity index	0	572 (78%)	32 (50%)	540 (81%)	< 0.001	32 (51%)	37 (59%)	0.174
	1	95 (13%)	21 (33%)	74 (11%)		21 (33%)	13 (20%)	
	2	41 (6%)	9 (14%)	32 (5%)		9 (14%)	8 (13%)	
	3	13 (2%)	1 (1%)	12 (2%)		1 (2%)	5 (8%)	
	≥ 4	11 (2%)	1 (1%)	10 (1%)		0	0	
BMI*	kg/m^2^	21.4 (19.0–23.8)	21.9 (18.8–24.4)	21.4 (19.1–23.7)	0.701	21.9 (18.8–24.4)	20.2 (18.4–22.9)	0.172
ASA・PS	1	356 (49%)	0	356 (53%)	< 0.001	0	4 (6%)	0.125
	2	306 (42%)	45 (70%)	261 (39%)		45 (71%)	43 (68%)	
	3	64 (8%)	19 (30%)	45 (7%)		18 (29%)	16 (26%)	
	4	5 (1%)	0	5 (1%)		0	0	
Malignancy	yes	29 (4%)	2 (3%)	27 (4%)	0.719	2 (3%)	8 (13%)	0.048
*Preoperative laboratory data*								
White blood cell*	/μL	12,300 (9500–15,400)	10,500 (7525–13,200)	12,600 (9600–15,600)	< 0.001	10,600 (7600–13,300)	9900 (6800–14,500)	0.725
Hemoglobin*	g/dL	13.8 (12.5–15.0)	12.6 (11.1–14.3)	13.9 (12.6–15.1)	< 0.001	12.6 (11.0–14.3)	13.3 (11.8–15.0)	0.147
Platelet*	× 10^4^/μl	23.0 (18.8–28.1)	22.0 (13.1–36.1)	19.5 (14.0–28.9)	0.455	21.7 (13.0–36.6)	19.0 (13.3–33.2)	0.742
Creatinine*	U/l	0.77 (0.64–0.94)	1.07 (0.88–1.70)	0.75 (0.63–0.91)	< 0.001	1.06 (0.88–1.66)	0.83 (0.68–1.10)	0.003
C-reactive protein*	mg/dl	1.9 (0.23–6.81)	1.99 (0.22–9.56)	1.89 (0.23–6.65)	0.706	1.90 (0.22–8.85)	1.17 (0.13–9.31)	0.934
PT(INR) *		1.09 (1.02–1.19)	1.13 (1.06–1.36)	1.09 (1.02–1.17)	< 0.001	1.13 (1.06–1.35)	1.08 (1.01–1.16)	0.005
APTT*	sec	28.8 (26.6–31.6)	29.8 (27.1–33.3)	28.7 (26.6–31.3)	0.020	29.7 (27.0–33.1)	28.0 (25.6–29.8)	0.003

AT: antithrombotic therapy, *: Median (Quartile), BMI: body mass index, ASA・PS: American Society of Anesthesiologists classification of physical status

PT(INR): prothrombin time-international normalized ratio, APTT: activated partial thromboplastin time

In the present study, median of the estimated blood loss with/without laparoscopic surgery or energy device were 56 mL versus 5 mL (*p* < 0.001) and 24 mL versus 5 mL (*p* < 0.001), respectively. Vessel-sealing devices and ultrasonically activated devices were used in 70 and 325 patients, respectively. In addition, vessel-sealing devices were frequently used in open surgery (n = 48). Therefore, these variables also should be adjusting. Perioperative outcomes between both groups after propensity score matching are shown in Table 3. There are no significant differences between the groups in each parameter, such as the number of laparoscopic surgeries, power device using, mean of the operating time, and estimated blood loss. The ratio of the number of blood transfusions during the perioperative period is also similar. There is no significant difference in grade II or greater postoperative morbidity in the AT and the Non-AT groups, respectively (14 [27%] vs. 16 [25%] patients, *p* = 0.676). Bleeding events developed in two (3.2%) and zero patients in the Non-AT and the AT groups, respectively (*p* = 0.094). Regarding other adverse events, neither stroke, embolic, nor thrombotic events occurred in the AT group patients, whereas stroke events occurred in one patient of the Non-AT group. Death within postoperative day 30 occurred in three (4.8%) and four (6.3%) patients in the AT group and the Non-AT group, respectively (*p* = 0.6969). There were also no significant differences between both groups in terms of postoperative stay (*p* = 0.409) and hospital death (*p* = 0.752).

**Table 3:** Perioperative outcomes of patients who underwent emergency gastrointestinal surgery

			No-matched cohort			Matched cohort		
Variables		n = 732	AT (n = 64)	Non-AT (n = 668)	*p* value	AT (n = 63)	Non-AT (n = 63)	*p* value
Laparoscopic surgery	Present	402 (55%)	14 (22%)	338 (58%)	< 0.001	14 (22%)	17 (27%)	0.501
Energy device	Present	395 (53%)	26 (41%)	369 (55%)	0.025	25 (40%)	25 (40%)	1.000
Operating time*	min	94 (71–124)	100 (62–128)	93.5 (71–124)	0.927	101 (62–129)	99 (74–131)	0.955
Estimated blood loss*	mL	10 (2–70)	40.5 (10–307)	8 (2–61)	< 0.001	36 (10–299)	53 (5–251)	0.466
Intra- and postoperative blood transfusion	Present	1 (0.2%)	11 (17%)	25 (4%)	< 0.001	11 (17%)	7 (11%)	0.309
Overall complications	Present	61 (8.3%)	21 (22%)	40 (6.0%)	< 0.001	14 (27%)	16 (25%)	0.676
Bleeding	Present	8 (1.1%)	3 (4.7%)	5 (0.75%)	0.025	0	2 (3.2%)	0.094
Stroke	Present	3 (0.4%)	3 (0.5%)	0	0.458	1 (1.6%)	0	0.238
Pulmonary	Present	12 (1.6%)	4 (6.3%)	8 (1.2%)	0.016	2 (3.2%)	3 (4.8%)	0.647
Renal/urological	Present	7 (1.0%)	1 (1.6%)	6 (0.9%)	0.630	2 (3.2%)	0	0.094
Surgical site infection	Present	29 (3.7%)	5 (7.8%)	24 (3.6%)	0.138	7 (11.1%)	5 (7.9%)	0.543
Anastomotic failure	Present	4 (0.5%)	1 (1.6%)	3 (0.5%)	0.335	2 (3.2%)	1 (1.6%)	0.555
Death within 30 days after surgery	Present	17 (2.3%)	5 (7.8%)	12 (1.8%)	0.013	3 (4.8%)	4 (6.3%)	0.697
Hospital stay after surgery*	Days	5 (4–10)	13 (9–22)	5 (4–9)	< 0.001	13 (9–22)	10 (7 -20)	0.409
Hospital death	Present	25 (3.4%)	6 (9.4%)	19 (2.8%)	0.020	5 (8%)	6 (10%)	0.752

*AT* antithrombotic therapy

*Median (Quartile)

We then assessed the associations between AT and intraoperative estimated blood loss (EBL), or postoperative bleeding complications. Intra-and post-operative blood transfusions were performed on 37 patients. The median blood loss of patients with or without blood transfusions was 492 mL (quartile: 171–903 mL) and 8 mL (quartile: 2–55 mL), respectively. Hence, we used 492 mL for the cut-off value of severe intraoperative bleeding for blood transfusions (n = 44).

Univariate and multivariate analyses for severe intraoperative bleeding and postoperative bleeding complications were performed and shown in Tables 4 and 5, respectively. In severe intraoperative bleeding analyses, sex (odds ratio 3.56, *p* = 0.001), appendectomy (odds ratio 0.15, *p* = 0.006), and colectomy (odds ratio 3.23, *p* = 0.012) were independent risk factors, and there was no association with antithrombotic drug use. Concerning postoperative bleeding complications analyses, antithrombotic drug use (odds ratio 1.08, *p* = 0.947), anticoagulant drug use (odds ratio 2.39, *p* = 0.584), and dual antithrombotic drug use (odds ratio 49.11, *p* = 0.058) were not independent risk factors similar to other variables.

**Table 4:** Univariate analysis for severe intraoperative bleeding (EBL ≥ 492 mL) and postoperative bleeding complication

		Severe intraoperative bleeding (EBL ≥ 492 mL)		Postoperative bleeding complication	
Variables	n = 732	Present	*p* value	Present	*p* value
*Age (years)*					
< 65	475	9 (1.9%)	< 0.001	1 (0.2%)	0.002
≥ 65	257	35 (13.6%)		7 (2.7%)	
*Sex*					
Female	332	12 (3.6%)	0.001	5 (1.5%)	0.327
Male	400	32 (8.0%)		3 (0.8%)	
*BMI (kg/m*^*2*^*)*					
< 22	333	18 (5.1%)	0.437	4 (1.2%)	0.282
≥ 22	259	18 (7.0%)		1 (0.4%)	
*Charlson comorbidity index*					
1–2	708	40 (5.7%)	0.061	6 (0.9%)	0.001
3–6	24	4 (16.7%)		2 (8.3%)	
*ASA・PS*					
1–2	663	26 (3.9%)	< 0.001	4 (0.6%)	< 0.001
3–5	69	18 (26.1%)		4 (5.8%)	
*Antithrombotic drugs*					
Absent	668	37 (5.5%)	0.113	5 (0.8%)	0.004
Present	64	7 (10.9%)		3 (4.7%)	
*Antiplatelet drugs*					
Absent	682	42 (5.8%)	0.291	6 (0.9%)	0.113
Present	50	2 (15.4%)		2 (3.9%)	
*Anticoagulant drugs*					
Absent	722	42 (5.8%)	0.223	6 (0.8%)	< 0.001
Present	12	2 (15.4%)		2 (15.4%)	
*Dual antithrombotic drugs*					
Absent	730	44(6.0%)	0.618	7 (1.0%)	< 0.001
Present	2	0		1 (50.0%)	
*Malignancy*					
Absent	700	38 (5.4%)	0.011	7 (1.0%)	0.258
Present	32	6 (18.8%)		1 (3.1%)	
*Appendectomy*					
Absent	297	41 (13.8%)	< 0.001	8 (2.7%)	0.001
Present	435	3 (0.69%)		0	
*Gastrectomy*					
Absent	728	42 (5.8%	0.014	8 (1.1%)	0.833
Present	4	2 (50%)		0	
*Patch repair*					
Absent	698	42 (6.0%)	0.974	7 (1%)	0.288
Present	34	2 (5.9%)		1 (2.9%)	
*Colectomy*					
Absent	669	25 (3.7%)	< 0.001	6 (0.9%)	0.096
Present	63	19 (30.2%)		2 (3.2%)	
*Intestinal surgery*					
Absent	584	30 (5.1%)	0.061	5 (0.9%)	0.221
Present	148	14 (9.5%)		3 (2.0%)	
*Cholecystectomy*					
Absent	697	41 (5.9%)	0.537	7 (1%)	0.304
Present	35	3 (8.6%)		1 (2.9%)	
*Drainage operation*					
Absent	7719	42 (5.8%)	0.223	7 (1.0%)	0.021
Present	13	2 (15.4%)		1 (7.7%)	
*Surgical approach*					
Laparoscopic surgery	404	6 (1.5%)	< 0.001	1 (0.3%)	0.015
Laparotomy	328	38 (11.6%)		7 (2.1%)	
*Energy device*					
Present	395	27 (6.8%)	0.307	3 (0.8%)	0.348
Absent	337	17 (5.0%)		5 (1.5%)

**Table 5:** Multivariate analysis for severe intraoperative bleeding (EBL ≥  492 mL) and postoperative bleeding complication

		Severe intraoperative bleeding (EBL ≥ 492 mL)			Postoperative bleeding complication		
Variable		Odds ratio	95% CI	*p* value	Odds ratio	95% CI	*p* value
Age (years)	< 65 vs. ≥ 65	2.16	0.86–5.41	0.101	2.76	0.34–75.61	0.377
Sex	Female vs. Male	3.56	1.65–7.66	0.001			
Charlson comorbidity index	1–2 vs. ≥ 3–6				5.63	0.69–34.42	0.097
ASA・PS	1–2 vs. ≥ 3-5	2.12	0.98–4.57	0.056	2.83	0.49–17.34	0.238
Antithrombotic drugs	Absent vs. Present				1.08	0.05–7.83	0.947
Anticoagulant drugs	Absent vs. Present				2.39	0.07–76.33	0.584
Dual antithrombotic drugs	Absent vs. Present				49.11	0.86–4780	0.058
Malignancy	Absent vs. Present	1.19	0.39–3.59	0.757			
Appendectomy	Absent vs. Present	0.15	0.04–0.58	0.006	2.81 × 10^-7	0–1.2 × 10^-27	0.063
Gastrectomy	Absent vs. Present	5.27	0.59–46.8	0.136			
Colectomy	Absent vs. Present	3.23	1.29–8.07	0.012			
Drainage operation	Absent vs. Present				3.97	0.18–36.17	0.315
Laparotomy	Absent vs. Present	2.34	0.88–6.24	0.090	1.24	0.16–25.64	0.850

*EBL* estimated blood loss, *ASA・PS* American Society of Anesthesiologists classification of physical status, *CI* confidence interval

## Discussion

Patients undergoing antithrombotic therapy (AT) have high risks of bleeding and thrombosis from their comorbidity [22, 23]. As those risks tend to be high when surgical intervention is required, several perioperative period protocols have been developed to prevent complications based on AT [5–7]. In contrast, the analysis of more than 400,000 surgical cases demonstrated that emergency procedures increase the risk of major postoperative morbidity and mortality [24]. Hence, the perioperative bleeding risk for patients undergoing AT may be expected to increase, making perioperative outcomes in an emergency setting a potential concern. Generally, as older people also have more comorbidities than young people, we hypothesize that perioperative complications will occur in patients with AT than those without AT.

Similar to other studies’ findings [8, 10], the patients in this study undergoing AT were older, have higher CCI, and poor ASA・PS than those who were not undergoing AT (Table 2). Advanced age is a well-known risk factor of cardiovascular disease, such as stroke [25] and coronary artery disease [26].

In using pre-matching data, there were no differences in operation time and estimated blood loss, whereas there were more Clavien-Dindo classifications of grade II or greater, longer postoperative hospital stay, and higher hospital death rate in the AT group than in the Non-AT group (Tables 2 and 3). A higher Carlson-comorbidity index and postoperative diagnosis are negative predictors of mortality, postoperative complications, and postoperative stay in older patients [27].

After adjusting these biases using the propensity score matching method, the estimated blood loss, perioperative blood transfusion, overall morbidity, and postoperative hospital stay were similar between the patients in both groups. Matsuoka et al. also demonstrated that antithrombotic drugs do not affect short surgical outcomes in their propensity score matching setting [28]. Additionally, Imamura et al. reported that there were no significant differences in blood loss, severe blood loss more than 100 mL, blood transfusion, and postoperative mortality for patients with or without antithrombotic therapy in emergency laparoscopic cholecystectomy [17]. These studies’ findings are line with our results, which may help surgeons in deciding the strategy for critical cases with antithrombotic therapy. Therefore, antithrombotic therapy does not affect perioperative short surgical outcomes in the situation of adjusting backgrounds.

We then evaluated the risk factors for the intraoperative severe bleeding needing blood transfusions in the all cohort. Although 492 mL is less than 750 mL [28] or 1000 mL [29] in previous studies, our results may be sustained with the use of new surgical instrumentations and techniques. Energy devices, including vessel-sealing devices and ultrasonically activated devices, have improved surgical outcomes in low anterior resection, liver transection, and hysterectomy [30–32]. These surgical techniques may be more effective in controlling intra- and postoperative bleeding in emergency surgery in patients undergoing antithrombotic therapy. In addition, there was no association between antithrombotic drug and blood transfusion in cases of severe intraoperative bleeding (Tables 4 and 5). In the elective setting, two randomized clinical studies recently demonstrated that bleeding and thrombotic events after elective non-cardiac surgery had no significant differences between patients with or without interruption of antiplatelet agents [33, 34]. Concerning abdominal surgery, several studies demonstrated the safety and feasibility of discontinued single aspirin or clopidogrel use in gastrointestinal surgery and colorectal resection [29, 35, 36]. These results, including ours, suggested that discontinuing antithrombotic drugs did not increase perioperative bleeding even in emergency surgery. On the other hand, a previous study reported that bleeding and mortality for emergency surgery were significantly higher than those for elective surgery in general surgical patients receiving warfarin [37]. Therefore, surgeons should pay more attention to manage the risk of intraoperative blood loss and reduce the need for blood transfusion in emergency gastrointestinal surgery in patients taking antithrombotic drugs.

Finally, we assessed the relationship between AT and postoperative bleeding. Here, there was a higher tendency of postoperative bleeding between patients taking and not taking dual antithrombotic drugs. The overall incidence of postoperative bleeding was low, and there were no statistical differences between the groups. The previous study reported that dual antiplatelet drugs increased postoperative bleeding [29, 38]. Therefore, as multiple antithrombotic drug use may increase postoperative bleeding complications in an emergency setting, careful postoperative management is necessary.

This retrospective study has several limitations. Several types of diseases were included, and the selection of surgical approach was not randomized. Antithrombotic therapy included many types and different generations of antiplatelet and anticoagulant agents. The total number of patients who underwent abdominal emergency surgery was relatively small in propensity score matching analyses. A comparison of the relationship between the patients with or without anticoagulant therapy in propensity score matched analysis was not performed due to their small number. Although administration of more than two anticoagulant drugs was a negative predictor for bleeding complications [29, 38], the present study included only two patients.

## Conclusion

This study indicated that intraoperative blood loss and postoperative complications related to emergency gastrointestinal surgery were not significantly affected by antithrombotic therapy.

## Data Availability

Data of the current study can be available from the corresponding author if requested.

## Abbreviations

AT: Antithrombotic therapy
EBL: Estimated blood loss
ASA・PS: Anesthesiologists classification of physical status
ROCcurve: Receiver operating characteristic
AUCs: Area under curves
UGI: Upper gastrointestinal
